# Advances in exploring activity cliffs

**DOI:** 10.1007/s10822-020-00315-z

**Published:** 2020-05-05

**Authors:** Dagmar Stumpfe, Huabin Hu, Jürgen Bajorath

**Affiliations:** Department of Life Science Informatics, B-IT, LIMES Program Unit Chemical Biology and Medicinal Chemistry, Rheinische Friedrich-Wilhelms-Universität, Endenicher Allee 19c, 53115 Bonn, Germany

**Keywords:** Activity cliff concept, Molecular similarity, Compound potency differences, Structure–activity relationships, Activity data analysis, Cliff categories

## Abstract

The activity cliff (AC) concept is of comparable relevance for medicinal chemistry and chemoinformatics. An AC is defined as a pair of structurally similar compounds with a large potency difference against a given target. In medicinal chemistry, ACs are of interest because they reveal small chemical changes with large potency effects, a concept referred to as structure–activity relationship (SAR) discontinuity. Computationally, ACs can be systematically identified, going far beyond individual compound series considered during lead optimization. Large-scale analysis of ACs has revealed characteristic features across many different compound activity classes. The way in which the molecular similarity and potency difference criteria have been addressed for defining ACs distinguishes between different generations of ACs and mirrors the evolution of the AC concept. We discuss different stages of this evolutionary path and highlight recent advances in AC research.

## Introduction

Activity cliffs (ACs) are of high interest in medicinal chemistry and chemical informatics. A Google Scholar search with the combined key words “activity cliff, medicinal chemistry, chemoinformatics” currently yields 1860 entries (947 since 2014). In addition, the six most cited papers with the term “activity cliff” in the title that are referred to in this Perspective have a cumulative count of 1496 citations. Of course, much of the practical work on ACs takes place in pharmaceutical research and is rarely reported.

In medicinal chemistry and chemoinformatics, activity cliffs (ACs) are defined as pairs or sets of structurally similar or analogous compounds that are active against the same target and have large potency differences [1–4]. Accordingly, ACs are the embodiment of structure–activity relationship (SAR) discontinuity, which limits compound activity predictions via quantitative SAR (QSAR) modeling [1], but provides important information for medicinal chemistry [2, 3].

Specifically, ACs reveal small chemical modifications with large potency effects that strongly influence or determine SARs. This information aids in compound optimization. However, during late stages of lead optimization, when high compound potency should be retained and other optimization-relevant properties need to be improved, the presence of steep SARs and ACs is often undesirable [5]. Hence, ACs might be viewed controversially in the practice of medicinal chemistry, depending on when they are encountered. However, regardless of whether encountering ACs is desirable or not, they generally have high SAR information content.

Compounds forming ACs are typically involved in multiple overlapping ACs. In fact, more than 90% of ACs available in compound data sets are formed by groups of structural analogs with varying potency, resulting in multiple ACs per compound [6]. These AC configurations can be explored in detail using network representations [6]. In AC networks, nodes represent compounds and edges pairwise AC relationships. The coordinated formation of ACs gives rise to clusters in AC networks [6]. These AC clusters contain much more SAR information than ACs analyzed as individual compound pairs. AC clusters often contain highly potent compounds having multiple weakly potent analogs, which results in densely connected nodes called hubs following network terminology [6]. In a different analysis, such AC hubs have also been designated AC generators [7], given their high propensity in forming ACs.

In compound data sets originating from different sources, for example, taken from different publications, as assembled in the ChEMBL database [8], ACs are likely detected with higher frequency than in individual compound series. However, these ACs are deprived of specific series-dependent optimization contexts [5]. While systematically identified ACs provide viable SAR information, they are more difficult to be appreciated by medicinal chemists than ACs detected in a specific optimization context.

Large-scale computational analysis of compound activity data has identified large numbers of ACs across currently available activity classes [9], yielding large volumes of SAR information. This information represents a valuable knowledge base for compound optimization, provided it can be efficiently and understandably communicated to medicinal chemists.

A practicing chemist might intuitively recognize and judge ACs while working on a particular compound series, based on experience. However, a systematic evaluation of ACs requires the unambiguous definition and consistent application of a molecular similarity criterion (i.e., when are two compounds “similar”?) and a potency difference criterion (i.e., when is a potency difference large enough to qualify as an AC?).

Setting these criteria and rationalizing their choice is at the core of the AC concept [2–4], as discussed in the following.

## Similarity and potency difference criteria for activity cliff analysis

### Compound similarity

In chemoinformatics, similarity for AC analysis has often been calculated on the basis of fingerprint descriptors and the Tanimoto metric [2, 9]. As a numerical similarity index, the Tanimoto coefficient [Tc] is straightforward to calculate. It ranges from 0 (for compound fingerprints without any overlap in bit settings) to 1 (identical fingerprints). For classifying compounds as similar, the choice of a similarity threshold value is required. Given that fingerprints are abstract (bit string) representations, many structural differences between compounds might lead to comparable Tc values. Structural relationships between AC candidate compounds detected on the basis of calculated similarity values are not limited to substitutions at given site(s). Rather, there might be multiple and different types of chemical modifications across these compounds. At a given Tc threshold value, a variety of whole-molecule similarity relationships are typically detected that may or may not be readily interpretable from a chemical viewpoint [2, 9]. Furthermore, calculated Tc values are dependent on the descriptors (fingerprints) that are used. Accordingly, generally applicable guidelines for the definition of Tc threshold values do not exist, and this also applies to other numerical similarity measures [9]. Because calculated similarity values are representation-dependent, it has been attempted to identify ACs that would be formed regardless of the chemoinformatic representations used, so-called consensus ACs [10].

**Fig. 1:**
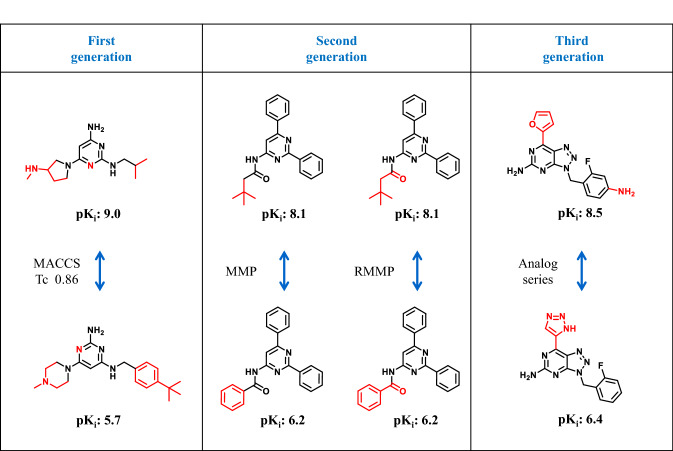
Exemplary activity cliffs. On the left, a MACCS-based AC is shown (Tc 0.86). Fingerprint-based ACs are first generation ACs. In the center, an MMP-cliff and a retrosynthetic version (RMMP-cliff) applying an activity class-dependent potency difference threshold are depicted (exemplary second generation ACs). On the right, an analog series-based AC with class-dependent potency difference threshold is shown (third generation AC). Further details are provided in the text. For all compounds, potency (pK_i_) values are reported and structural differences are highlighted in red. From the left to the right, AC targets were the histamine H4, adenosine A1, and adenosine A2a receptor, respectively

As an alternative to numerical similarity metrics, substructure-based similarity measures are also applicable for AC definition and identification [3, 9]. The use of substructure-based similarity criteria does not require threshold values. The basic principle is that two compounds either contain a given substructure or not, yielding a binary (yes/no) readout of similarity [9]. Of course, as a similarity criterion, substructures can be defined in many different ways [9, 11] and there is no ultimate answer which substructure formalism might best be applied for AC assessment. A convenient way of algorithmically establishing substructure relationships, without the need to pre-define substructures, is the calculation of matched molecular pairs (MMPs) [12]. An MMP is defined as a pair of compounds that are only distinguished by a structural modification at a single site [12, 13]. If appropriate size restrictions for the core structure and substituent fragment are introduced, the resulting MMPs are essentially confined to pairs of structural analogs [14], providing the similarity criterion for MMP-cliffs [14], one of our preferred substructure-based AC definitions. For medicinal chemistry applications, the MMP-cliff formalism has been further refined by generating MMP fragments on the basis of retrosynthetic rules, yielding RMMP-cliffs [15]. By definition, MMP- and RMMP-cliffs are limited to substitutions at a single site, which accounts for a subset of structural relationships in analog series where substitutions at more than one site often occur. Therefore, as an extension of the MMP-cliff concept, analog pairs might be systematically enumerated for given or computationally identified analog series [16], which makes it possible to identify ACs with multiple substitution sites originating from the same series [17]. Figure 1 displays representative examples of fingerprint-, substructure-, and analog series-based ACs.

ACs formed by structural isomers (iso-ACs) [18] and chirality ACs [11] capture overall smallest structural variations leading to AC formation. Iso-ACs contain the same substituent at two different sites in a compound while compounds forming chirality cliffs are only distinguished by different chirality at a given stereocenter. The formation of iso-ACs can also be combined with the detection of MMP relationships, thereby establishing a category of ACs that is based upon a combination of different similarity criteria [18]. Furthermore, chirality ACs might also be represented using different chirality-depending chemical descriptors, yielding so-called chiral cliffs [19], which have been used to study ACs formed by enantiomers tested in the same assays [19].

### Potency difference thresholds

The assessment of potency differences that are relevant for AC formation relies on comparing experimental values. As potency measurements, the use of (in theory) assay-independent equilibrium or dissociation constants (K_i_ or K_D_ values, respectively) is generally preferred to ensure high accuracy of AC assignments. Although ACs can formally also be assessed as a continuum of pairs of compounds with increasing potency differences [20], the application of a constant potency difference threshold has largely dominated AC analysis and the systematic search for ACs in compound databases [2, 3]. A constant potency difference threshold should be larger than most pairwise potency differences in analog series or compound activity classes and statistically significant. An at least 100-fold difference in potency has frequently been applied in AC analysis [2–4]. The application of a constant potency difference threshold enables the computational search for ACs across different activity classes. Requiring an at least 100-fold potency difference for AC formation typically limits ACs to ~ 5% of all qualifying pairs of structurally similar compounds [2, 3]. However, a constant threshold does not take activity class-dependent differences in compound potency distributions into account.

**Fig. 2:**
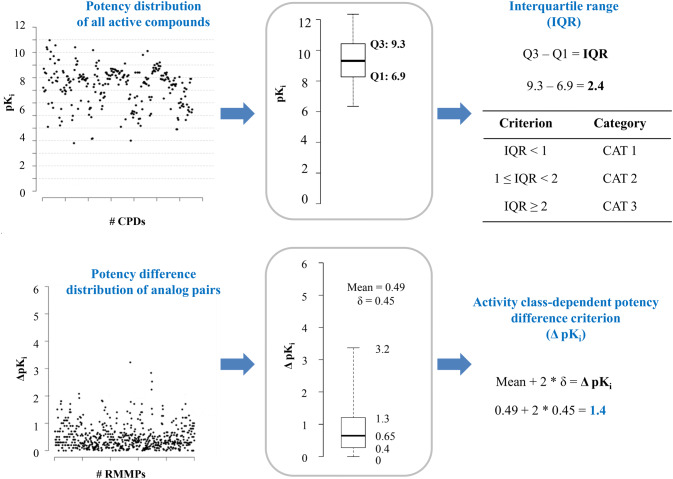
Activity class-dependent potency difference thresholds. The compound potency distribution for neurokinin 1 receptor ligands (top left) is represented in a boxplot (center) and the interquartile range (IQR) is determined (right). On the basis of the IQR, activity classes are assigned to different categories (IQR < 1; CAT 2: 1 ≤ IQR < 2; CAT 3: IQR ≥ 2) and only classes of CAT 2 or 3 are subjected to AC analysis. At the bottom, the corresponding potency difference distribution of RMMPs is displayed (left). From the mean and standard deviation (σ) of the distribution (center), the activity class-dependent potency difference threshold for AC formation is calculated as the mean plus two σ (ΔpK_i_ = 1.4) (right)

Compound potency distributions in activity classes vary greatly and so do compound similarity relationships [21]. Accordingly, AC formation should best be considered in an activity class-dependent manner. The derivation of class-dependent potency difference thresholds further refines AC analysis for specific biological activities. Therefore, statistically significant activity class-dependent potency difference thresholds have been systematically investigated (Fig. 2). On the basis of statistical considerations, class-dependent thresholds were ultimately determined as the mean of the compound pair-based potency difference distribution plus two standard deviations [22] (Fig. 2). The introduction of class-dependent thresholds changes global AC statistics across bioactive compounds, as expected and further discussed below.

## Different generations of activity cliffs

The way in which similarity and potency difference criteria are addressed and combined mirrors the evolution of the AC concept. Considering this evolutional path, we have recently distinguished between three generations of molecular graph-based (two-dimensional; 2D) ACs [4, 23], as illustrated in Fig. 1.

According to this classification scheme, “first generation” ACs are characterized by the use of numerical or substructure-based similarity measures and application of a constant potency difference threshold across all activity classes.

In addition, “second generation” ACs result from the application of the (R)MMP-cliff formalism, capturing structural analogs with single substitution sites, and variable activity class-dependent potency difference thresholds.

Furthermore, “third generation” ACs are formed by analogs from the same series, i.e. analog pairs with single or multiple substitution sites, applying activity class-dependent potency difference thresholds.

As a rule of thumb, the chemical interpretability and SAR information content of ACs increases over these generations.

## Three-dimensional activity cliffs

Importantly, the assessment of ACs is not limited to molecular graph-based representations. ACs can also be studied in three dimensions. Currently, there is no crystallographic study reported that has set out to determine structures of complexes of a given target with ligands forming ACs identified on the basis of molecular graphs. However, ACs can be also defined on the basis of three-dimensional (3D) structures of protein–ligand complexes, leading to the identification so-called 3D-cliffs [24, 25]. Therefore, crystallographic complexes of compounds bound to the same target protein must be identified, target structures from different complexes carefully superposed, and binding poses of compounds transferred to a reference complex. For the resulting target-based ligand overlays, 3D similarity of ligand binding modes is calculated in a pairwise manner and related to potency differences obtained from literature sources [24]. Different numerical 3D similarity functions are available to quantify shape and/or molecular property overlap, for example, using atomic property density functions [26, 27]. As with any numerical similarity measure, threshold values for 3D similarity must be pre-defined (e.g., 85% binding mode similarity).

**Fig. 3:**
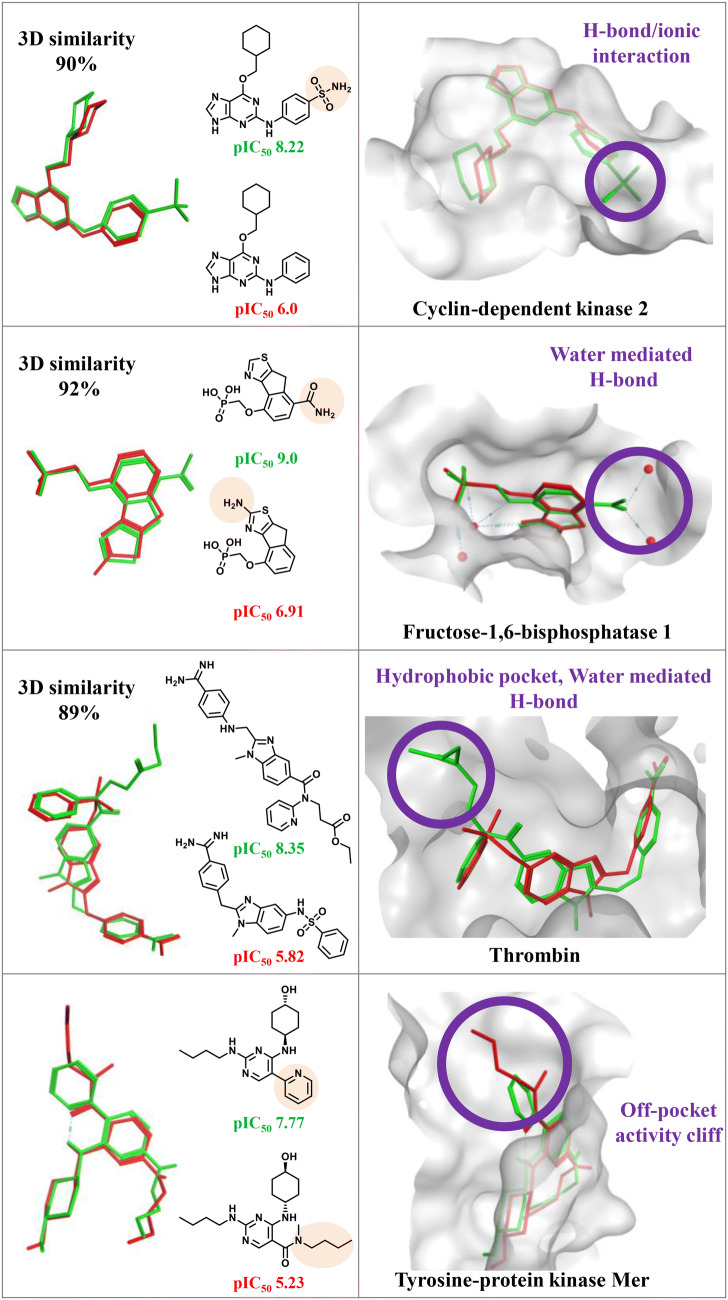
Three-dimensional activity cliffs. Shown are three exemplary 3D-cliffs where ligands are distinguished by different types of interactions. Bound conformations of highly and weakly potent cliff compounds are colored green and red, respectively. In addition, an exemplary off-pocket cliff according to reference 28 is shown at the bottom

3D-cliffs are attractive because they reveal differences in ligand-target interactions that might be responsible for AC formation. Accordingly, 3D-cliffs have been classified according to different interactions that distinguish between weakly and highly potent cliff compounds such as, among others, the presence or absence of specific hydrogen bonds or hydrophobic substituents (filling complementary hydrophobic pockets in binding sites) [24]. Figure 3 shows different examples of 3D-cliffs. Hypotheses concerning critical interactions derived from X-ray structures and 3D-cliffs are still subject to experimental evaluation and confirmation. Regardless, 3D-cliffs provide valuable information for SAR exploration and drug design. Importantly, insights obtained from 3D-cliffs are limited to differences between short-range interactions revealed by X-ray structures, which represent the endpoint of binding events. Moreover, X-ray structures provide an incomplete picture of binding processes, which involve a variety of factors that influence binding such as solvation/desolvation energies or entropy changes associated with or going beyond the hydrophobic effect. Therefore, there are frequent examples of 3D-cliffs that cannot be rationalized on the basis of differences between ligand-receptor interactions revealed by X-ray structures [24].

In a recent study, so-called off-pockets cliffs were identified where distinguishing structural modifications of AC compounds in X-ray structures mapped to the solvent environment and were not involved in ligand-target interactions [28]. Exemplary off-pocket cliffs were then analyzed computationally via molecular dynamics simulations and Markov state modeling. The results indicated that solvent-exposed compound modifications with apparent potency impact often changed the dynamics of ligand-target interactions and solvation, inducing propagating effects on buried moieties of bound ligands that were likely to modulate the strength of interactions [28]. An exemplary off-pocket cliff is shown in Fig. 3 (bottom).

It should be noted that the analysis of graph-based (2D) ACs and 3D-ACs is not mutually exclusive. Thus far, we have not identified 3D-cliffs that were not detectable on the basis of molecular graph comparison. The analysis of ACs in two and three dimensions can be combined in different ways. For example, SAR information provided by 3D-cliffs can be further increased by identifying active structural analogs of cliff compounds through database searching [25]. This facilitates an extension of 3D-cliffs through the addition of 2D analogs that might form additional 3D/2D-ACs [25]. In a recent systematic analysis of X-ray structures of small molecules in complexes with human targets from the Protein Data Bank [29], a total of 630 3D-cliffs were identified for which high-confidence activity data [30] were available [24]. These ACs covered 61 human target proteins. A systematic search identified 1980 analogs of 268 3D-cliff compounds in ChEMBL for which high-confidence activity data were also available. These analogs extended 414 3D-cliffs that were active against 50 human targets [25]. Hence, there is a substantial body of structural AC information available, which can be complemented through analog searching. Going a step further, 2D- and 3D-ACs with shared compounds have been assessed using similarity calculations based upon molecular graph-derived fingerprints as well as 3D interaction fingerprints. 3D-ACs established on the basis of interaction fingerprints were designated interaction cliffs [31]. For kinase inhibitors and their X-ray complexes, only about a quarter of detected 2D-ACs could be reproduced on the basis of calculated 3D interaction similarity [31]. Nonetheless, interaction similarity provides an alternative to other 3D similarity measures and is particularly suitable for uncovering interaction hot spots across 3D-ACs for a given target family.

## Identifying activity cliffs on a large scale

Computational compound representations and well-defined structural similarity and potency difference criteria enable the systematic search for ACs across the current spectrum of bioactive compounds, going far beyond the analysis of individual compound series. In this section, we summarize results of recent large-scale investigations of different generations of ACs, as defined above.

### First generation ACs

A systematic survey of these ACs was reported in 2015 on the basis of ChEMBL release 20 [32]. From ChEMBL, 48,244 unique compounds with activity against 746 targets were extracted for which high-confidence activity data [30] including K_i_ values were available. For these 746 activity classes (also termed target sets), first generation ACs (ΔpK_i_ ≥ 2) were determined using two fingerprints of different design, MACCS structural keys [33] and the extended connectivity fingerprint with bond diameter 4 (ECFP4) [34], as well as applying the MMP formalism. The results are summarized in Table 1 (left). Nearly twice as many fingerprint-based ACs (ECFP4 or MACCS) than MMP-cliffs were identified. Specifically, there were 31,975 ECFP4- and 34,813 MACCS-based ACs compared to 17,111 MMP-cliffs. Thus, MMP-cliffs were a structurally more conservative representation of ACs. The numbers of AC-forming compounds were very similar for the fingerprint-based ACs (16,186 and 16,614 corresponding to 33.6% and 34.5%, respectively) compared to a reduced number of 11,030 compounds (22.9%) participating in MMP-cliffs.

**Table 1:** First generation activity cliff statistics

	ChEMBL 20			ChEMBL 25		
	ECFP4	MMP	MACCS	ECFP4	MMP	MACCS
# ACs	31,975	17,111	34,813	61,524	18,749	79,338
# QPs	624,420	384,725	564,071	1,106,985	354,751	1,076,185
% ACs	5.1%	4.8%	6.2%	5.9%	4.6%	6.9%
# AC compounds	16,186	11,030	16,614	24,657	9590	26,223
% AC compounds	33.6%	22.9%	34.5%	37.5%	14.6%	39.9%

“# ACs” reports the total number of activity cliffs for each molecular representation and “# QPs” gives the total number of qualifying compound pairs meeting the respective similarity criteria for AC formation. “% ACs” reports the percentage of all QPs that formed ACs. In addition, “# AC compounds” gives the total number of compounds involved in the formation of ACs and “% AC compounds” the proportion of all compounds forming ACs

For comparison, we report here up-to-date statistics for first generation ACs using ChEMBL release 25. To these ends, only activity classes that contained at least 100 compounds were considered. A total of 65,766 unique compounds having high-confidence activity data with K_i_ values for 192 targets were obtained. Notably, compared to MMP-cliffs, numbers of fingerprint-based ACs significantly increased from twofold (ChEMBL 20) to three- to fourfold (ChEMBL 25). While 18,749 MMP-cliffs were identified, 61,524 ECFP4- and 79,338 MACCS-based ACs were detected. However, the number of qualifying compound pairs, i.e., pairs exceeding a Tc threshold value of 0.55 (ECFP4) or 0.85 (MACCS), dramatically increased to more than a million. Despite this unprecedented increase in qualifying compound pairs, the proportion of ACs remained essentially constant, with 5.9% (ECFP4), 6.9% (MACCS), and 4.6% (MMP-cliffs), consistent with earlier findings. This unexpectedly large increase in the number of compound pairs then resulted in the three- to fourfold increase in fingerprint-based first generation ACs over the course of only four to five years.

### Second generation ACs

The first systematic search for second generation ACs was carried out in ChEMBL release 23. A total of 212 activity classes with available K_i_ measurements and potency value distributions with potential for AC formation [21] were identified that yielded a total of 16,096 class-dependent RMMP-cliffs [22]. The majority of activity class-dependent potency difference thresholds fell into the range 1 ≤ ΔpK_i_ ≤ 2.5. When a constant potency difference threshold of ΔpK_i_ ≥ 2 was applied across all activity classes, 11,773 RMMP-cliffs were identified in 195 classes [22]. The comparison showed that the application of class-dependent potency difference threshold led to the formation of more ACs covering more targets than a generally applied constant potency difference threshold of comparable magnitude. In addition, given the statistically grounded definition of class-dependent potency difference thresholds, ACs were more evenly distributed across different activity classes. Furthermore, second generation ACs were also defined taking compounds into account that were confirmed to be inactive (rather than weakly potent) in screening assays available in PubChem [35]. For eight of 73 activity classes with available screening data, only 145 additional RMMP-cliffs involving inactive compounds were identified [36]. Hence, taking screening data into account, there only was a small increase in the number of second generation ACs.

**Fig. 4:**
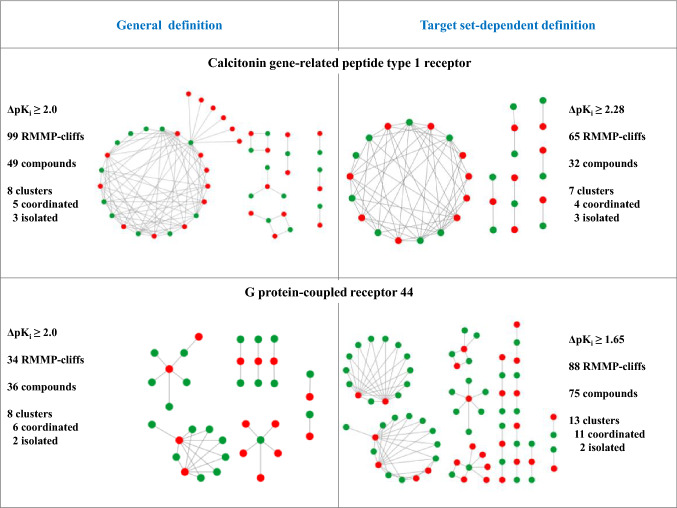
Activity cliff networks. For two activity classes, RMMP-cliff networks are shown. Nodes represent compounds and edges pairwise ACs. Highly and weakly potent cliff partners are colored green and red, respectively. Networks on the left and right were generated applying are constant potency difference threshold (ΔpK_i_ ≥ 2) and an activity class-dependent threshold (as specified), respectively. In each case, the number of ACs, participating compounds, and AC clusters are reported. Clusters containing coordinated ACs are distinguished from clusters formed by isolated ACs

In Fig. 4, small exemplary AC networks are shown for a generally applied potency difference criterion and compared to corresponding networks based on activity class-dependent potency difference thresholds. In the example at the top, the number of RMMP-cliffs decreased from 99 to 65 when the class-dependent threshold was applied. However, SAR information was essentially retained since seven of eight AC clusters remained. By contrast, in the example at the bottom, application of the class-dependent threshold increased the number of RMMP-cliffs from 34 to 88. Here, the gain of 54 additional ACs gave rise to the formation of five new AC clusters with different structural contexts and thus led to a substantial increase in SAR information.

A large collection of second generation ACs resulting from our systematic analysis has been made publicly available [36].

### Third generation ACs

A systematic search in ChEMBL release 24.1 identified 16,454 analog series-based ACs applying class-dependent potency difference thresholds [17]. However, with 4205 instances, only 25.6% of these third generation ACs were multi-site cliffs that included 3805 (90.5%) dual-site (ds-) ACs [17]. Hence, most third generation ACs only contained a single substitution site and ds-ACs clearly dominated the distribution of multi-site ACs.

To complement recent AC surveys, we have also determined the growth of second and third generation ACs over time, covering a number of years prior to their formal introduction [4]. From ChEMBL release 25, all compounds with available high-confidence activity data, K_i_ values, and an explicitly reported year of publication or release were systematically extracted. In 2018, 65,766 qualifying compounds with activity against 192 targets were available. Beginning with this data set, annually added compound increments were traced back to 2009 and for each year, cumulative data subsets were generated (containing compounds released up to and including that year). For each activity class and year, an increment of at least 100 compounds was required.

Then, the number of second and third generation ACs was determined for each year. Results for six of 10 years are summarized in Table 2. The number of second generation RMMP-cliffs increased from 3134 in 2009 to 9787 in 2018. Hence, the number of ACs increased around three-fold and the number of activity classes with ACs nearly doubled from 91 in 2009 to 170 in 2018. The proportions of second generation ACs among qualifying compound pairs remained constant at ~ 5.0% over the course of 10 years. In addition, third generation ACs increased from 5516 occurrences in 108 activity classes in 2009 to 16,957 in 192 classes in 2018. However, the proportion of analog pairs forming ACs also remained constant at ~ 5.0%. Furthermore, there was a slight increase in the proportion of multi-site ACs compared to single-site ACs. The comparison shows that we continue to experience substantial growth in AC information over time, with an essentially constant proportion of new bioactive compounds and pairs participating in AC formation.

**Table 2:** Monitoring second and third generation activity cliffs over time

	2009	2010	2012	2014	2016	2018
Second generation activity cliffs (RMMP-cliffs)						
# Targets	91	103	123	145	163	170
# ACs	3134	4057	5041	7808	9409	9787
% ACs	5.0%	4.9%	5.0%	5.0%	5.0%	5.0%
Third generation activity cliffs (RMMP-cliffs)						
# Targets	108	121	144	171	184	192
# ACs	5516	7031	8787	13,602	16,168	16,957
% ACs	5.0%	4.9%	5.0%	4.9%	5.0%	5.0%
# ss-ACs	4080	4836	5829	9994	12,005	12,326
# ds-ACs	1319	2061	2729	3302	3811	4214
% ss-ACs	4.4%	4.2%	4.1%	4.2%	4.3%	4.2%
% ds-ACs	8.3%	7.8%	8.7%	8.9%	9.1%	9.4%

“# Targets” reports the number of activity classes and “# ACs” the total number of activity cliffs for each year. “% ACs” gives the proportion of ACs among qualifying compound pairs. In addition, “# ss-ACs” and “# ds-ACs” separately report the number of third generation single-site and dual-site ACs and “% ss-ACs” and “% ds-ACs” the proportion of ss-ACs and ds-ACs among qualifying compound pairs

## Prediction of activity cliffs

In addition to identifying ACs on the basis of large-scale activity data analysis, attempts have also been made to predict ACs in compound data sets. The first study reported applied random forest (RF) modeling in combination with descriptor aggregation and SAR analysis functions to predict compounds that would form ACs with given ones [37]. The resulting models were predictive but their accuracy was limited. Higher prediction accuracy was achieved in distinguishing between pairs of analogs that formed or did not form ACs. These predictions were facilitated using support vector machine (SVM) classification with especially designed kernel functions that captured structural differences between paired compounds [38]. SVM models were trained to associate structural modifications captured in MMPs with potency differences between MMP-forming compounds applying a constant threshold for AC formation and used to predict MMP-cliffs [38]. Furthermore, support vector regression (SVR) models were built to quantitatively predict MMP-associated potency changes, yielding accurate predictions for a variety of activity classes [39]. Here, SVR models reached higher performance levels than RF regression models. This approach enabled the prediction of ACs of varying magnitude. However, as mentioned above, potency predictions for AC compounds using QSAR approaches are generally difficult, regardless of descriptors and methods used [40]. This is the case because QSAR modeling is principally based on the presence of SAR continuity when gradual changes in molecular structure cause small to moderate changes in potency.

SVM and SVR modeling were also applied to predict ACs represented using the condensed graph of reaction formalism (adapted from chemical reaction modeling) or descriptor recombination (adapted from QSPR modeling of non-additive mixtures) [41]. These representations encoded ACs as single feature vectors, hence alleviating the need to use special kernel functions for SVR or SVR modeling, but yielded comparable prediction accuracy. Furthermore, chiral cliffs were also predicted among pairs of enantiomers using logistic regression, RF, and gradient boosting on the basis of vectors of various chirality-sensitive molecular descriptors [19]. The resulting models produced accurate predictions, with gradient boosting achieving slightly higher accuracy than RF [19].

Few attempts have been made to predict 3D-cliffs and the compound potency differences they represent. Different docking techniques and scoring schemes were applied to investigate 3D-cliffs, approximate potency differences between cliff-forming compounds, and predict compounds that would form ACs with similar ones having experimentally known or hypothetical binding models [42]. In addition, potency differences between 3D-cliff compounds have been predicted using free energy perturbation calculations, frequently with an accuracy of close to or within one order of magnitude compared to experiment [43]. Although only very few structure-based AC predictions have been reported so far, they have produced some promising results. For free energy perturbation methods, high-confidence 3D-ACs provide excellent test cases.

Notably, in independent studies, machine learning on the basis of conceptually different AC representations often reached prediction accuracy at or even beyond the 80% level. Accurate predictions indicated that ACs systematically capture structural patterns responsible for large potency variations of compounds with specific biological activities, hence reinforcing the utility of ACs for SAR exploration from a different perspective.

## Emphasis on medicinal chemistry applications

**Fig. 5:**
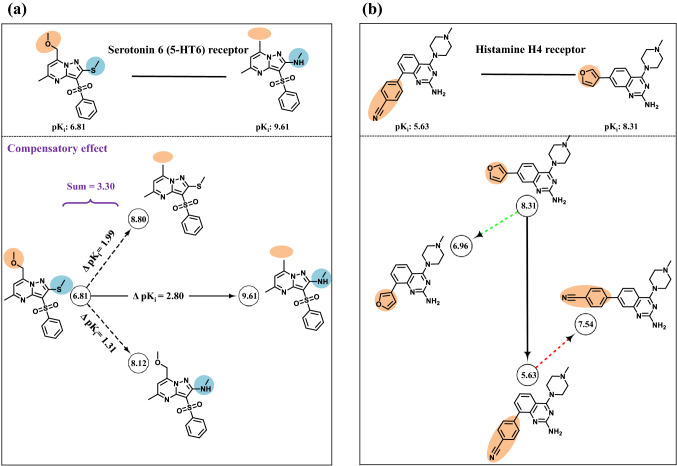
Dual-site activity cliffs and single-site analogs. **a** Shown is an exemplary ds-AC together with both single-site analogs displaying a compensatory potency effect on AC formation. Structural modifications at different sites are colored orange and blue, respectively, and pK_i_ values are reported in circles. **b** Shown is an exemplary ds-AC with two structural isomers (connected with the highly and weakly potent ds-AC compound through dashed green and red arrows, respectively). Structural modifications are highlighted in orange and pK_i_ are reported in circles

Systematic identification and prediction of ACs falls into the methodological arena of chemoinformatics. Equally important are computational studies on ACs that impact medicinal chemistry. For example, ds-ACs (containing substitutions at two sites) can be further analyzed and interpreted by searching for analogs that capture chemical modifications at individual sites and make it possible to evaluate their contribution to AC formation (single-site analogs). For 297 of the ds-ACs reported, we have identified two single-site analogs that contained the individual substitutions [17]. If both single-site analogs are available for a given ds-AC, a new four-compound data structure is obtained. Comparing the potency of ds-AC compounds with associated single-site analogs identified a number of ds-ACs whose potency differences were accounted for by a single substitution (termed redundant ds-ACs). In addition, additive, synergistic, and compensatory potency effects of substitutions in ds-ACs were detected [17], which rationalized ds-AC formation and provided additional SAR information. Figure 5a shows an exemplary ds-AC with both single-site analogs displaying a compensatory potency effect. In addition, Fig. 5b shows another ds-AC complemented with structural isomers of ds-AC compounds, which also aided in rationalizing AC formation.

**Fig. 6:**
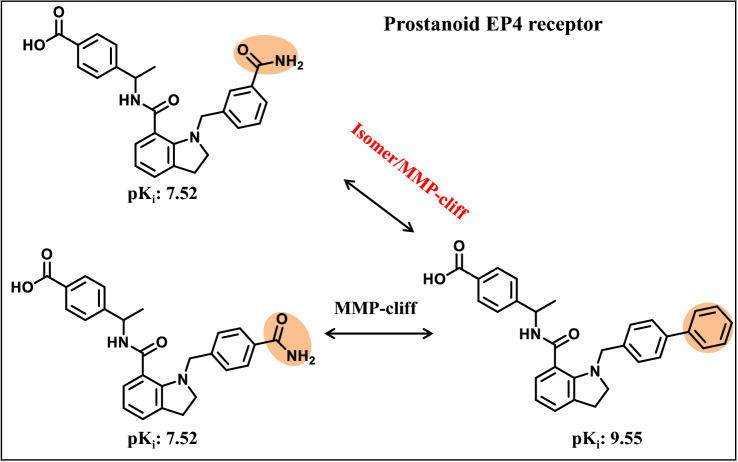
Isomer/MMP-cliffs. Shown is an exemplary isomer/MMP-cliff with activity against the prostanoid EP4 receptor. A structural isomer of the weakly potent MMP-cliff compound was identified forming an additional AC with the highly potent cliff compound

Similar considerations are applicable to isomer/MMP-cliffs combining two substructure-based similarity criteria [18]. In a recent study, 597 isomer/MMP-cliffs were identified by first identifying MMP-cliffs and then searching for structural isomers of MMP-cliff compounds [18]. Figure 6 shows an exemplary isomer/MMP-cliff arrangement.

**Fig. 7:**
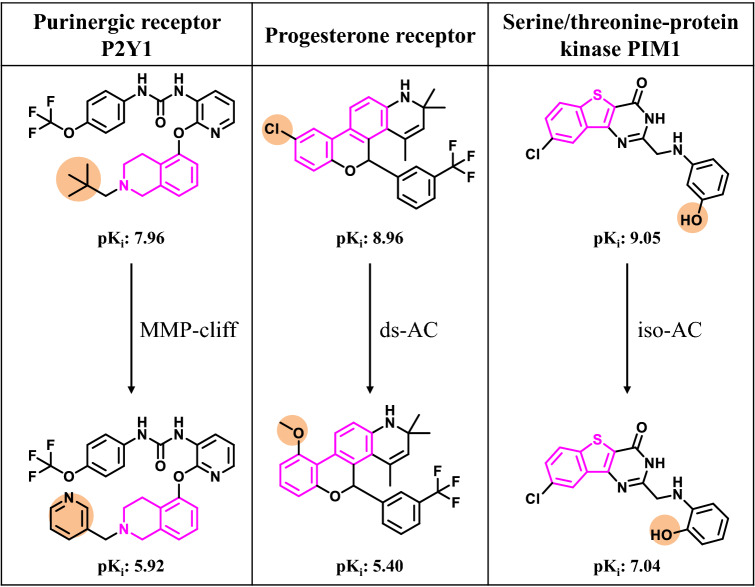
Activity cliffs with privileged substructures. Different ACs containing PSs are shown. Substituents and the PS are colored in orange and pink, respectively. For each AC, the target name and potency values are provided

Another application of immediate relevance for medicinal chemistry is the exploration of ACs formed by compounds containing privileged substructures (PSs) [44]. These structures are often found in compounds with preferential activity against individual target families and are thus of high interest for compound design in medicinal chemistry [44, 45]. Recently, a systematic search has been carried out for ACs with PSs (PS-ACs) [46]. For 24 PSs found in at least 100 ChEMBL compounds, a total of 15,919 PS-ACs were identified, accounting for 46.7% of all detected ACs. Exemplary PS-ACs are shown in Fig. 7. Among them, PS-containing MMP-cliffs dominated the distribution, followed by ds-ACs, and iso-ACs (with 12,150, 3544, and 225 instances, respectively). On the basis of molecular property analysis (including, among others, logP and ligand efficiency), there were no statistically significant differences between ACs with and without PSs. However, for individual PSs, substantial differences in the frequency of PS-AC formation were detected. In addition, many PS-containing compounds were on average more frequently involved in AC formation than other bioactive compounds. If one considers ACs as an indicator of SAR responses, PSs are likely to display varying SAR characteristics in different structural environments, lending further support for their consideration as target family-directed scaffolds in medicinal chemistry.

## Conclusions and perspective

ACs were first discussed in the computational and medicinal chemistry literature about 25 years ago. The way in which molecular similarity and potency difference criteria are defined plays an important role for the assessment, interpretation, and utility of ACs and mirrors the evolution of the AC concept. Much of our current knowledge and understanding of ACs resulted from chemoinformatics, in particular, large-scale analysis of compound activity data applying different AC definitions. However, the major application areas for AC information are SAR analysis and compound optimization in medicinal chemistry. In our experience, AC information from external sources is still underutilized in the practice of medicinal chemistry. Any hit-to-lead and lead optimization campaign should take already available SAR information from other compounds active against the target of interest into consideration. However, communicating this information to medicinal chemists in a meaningful way is challenging, given that their natural focus is on sequential optimization efforts and individual compound series. We envision that expert systems might be required to retrieve all ACs from a database that involve structurally related compounds for a series of interest having the same activity. Such on-demand access to AC information might help to close the gap between chemoinformatic analysis and the practice of medicinal chemistry.

In our view, major steps forward in AC research have been the recent rationalization and application of activity class-dependent potency difference thresholds and the study of multi-site ACs (that these works originated from our group is by coincidence and not to be regarded as a promotional effort). The introduction of variable potency difference thresholds pays tribute to varying SAR characteristics of compound activity classes and the fact that ACs for a given target might not at all be comparable to ACs for another. Class-dependent potency difference thresholds render AC analysis dependent on compound potency distributions for given targets and lead to a balanced distribution of ACs over different activity classes. In addition, multi-site ACs enable the investigation and comparison of contributions of individual substitution sites to AC formation, as shown herein, which further improves the interpretability of ACs and their relevance for SAR exploration.

We also note that different extensions of the AC concept have been introduced over time such as 3D-cliffs and interaction cliffs discussed herein or promiscuity cliffs (PC) [47, 48], which we are particularly interested in. In PCs, compound potency differences are replaced with the difference in the number of target annotations of cliff compounds. Accordingly, PCs and further extended data structures generated on the basis of PCs [48] are of interest for systematically exploring structure-promiscuity relationships and better understanding the basis of multi-target activity of small molecules.

Where do we go from here? The AC concept will certainly further evolve and new AC variants will likely be considered. In addition, new strategies for predicting ACs and identifying structural features that might be signatures of potent compounds across different activity classes should be interesting topics for future research, with implications for drug design. Furthermore, as X-ray data continue to grow significantly, there are opportunities to further extend and refine the study of 3D-ACs, which will likely yield guidelines for structure-based design. It is also noted that 3D-ACs provide attractive test cases for scoring functions used in docking or free energy methods. In some ways, it is surprising that only very few studies have been reported to date that explicitly make use of 3D-cliffs for testing and calibrating quantitative computational approaches for activity or free energy predictions. Perhaps this might be attributed to difficulties in reliably predicting potency values or relative free energies of binding. Regardless, one would hope for more studies addressing such prediction tasks on the basis of 3D-ACs. Many potential test cases have been made publicly available. Last but not least, how to improve the utility of large volumes of AC information for medicinal chemistry continues to be an essentially open yet critical question. As we have gained substantial knowledge about ACs and their characteristics by now, translating AC information into practically applicable compound design strategies will without doubt be a major goal going forward.
